# Association of SPOP Expression with the Immune Response to *Salmonella* Infection in Chickens

**DOI:** 10.3390/ani10020307

**Published:** 2020-02-14

**Authors:** Fei Wang, Qinghe Li, Qiao Wang, Maiqing Zheng, Jie Wen, Guiping Zhao

**Affiliations:** 1 Institute of Animal Sciences, Chinese Academy of Agricultural Sciences, Beijing 100193, China; feiwang2840@163.com (F.W.); qli2014@126.com (Q.L.); wangqiao@caas.cn (Q.W.); zhengmaiqing@caas.cn (M.Z.); wenjie@caas.cn (J.W.); 2 State Key Laboratory of Animal Nutrition, Beijing 100193, China; 3 School of Life Science and Engineering, Foshan University, Foshan 528225, China

**Keywords:** SPOP, *Salmonella*, immune response, IL-1β, IL-8, IgA

## Abstract

**Simple Summary:**

*Salmonella* frequently causes human illness via the consumption of contaminated meat or eggs. At present, studies about how the host immune response against *Salmonella* is regulated are limited. Speckle-type POZ (poxvirus and zinc finger) protein (SPOP) is a specific adaptor of Cul3-based ubiquitin ligase, which catalyzes the ubiquitination and degrades the substrates. However, its role in the immune response is still unknown. Therefore, this study measured expression of SPOP and the proinflammatory cytokines interleukin-1β and interleukin-8 in chicken macrophage cells stimulated with a bacterial substitute and assessed their relationship using the quantitative polymerase chain reaction. We then validated the results in chickens infected with *Salmonella*. Notably, SPOP expression gradually decreased and then gradually increased in cells after challenging the bacterial substitute, indicating its potential involvement in the regulation of the immune response. Additionally, SPOP expression was negatively correlated with expression of interleukin 1β and interleukin-8 both in vivo and in vitro. More importantly, SPOP expression was related to immunoglobulin (Ig) A production and bacterial loads in chickens infected with *Salmonella.* These results indicate that SPOP could be a potential marker of the immune response in chickens.

**Abstract:**

Salmonellosis is a zoonosis that is not only harmful to the health of poultry but also poses a threat to human health. Although many measures have been put in place to reduce morbidity, they have not provided satisfactory results. Therefore, it is necessary to clarify the immune mechanisms involved in improving the resistance of chickens against *Salmonella*. BTB (Broad-complex Tramtrack and Bric-a-brac) Speckle-type POZ (poxvirus and zinc finger) protein (SPOP) regulates protein expression by promoting substrate ubiquitination and degradation. The correlation between SPOP expression and the immune response has not been fully described. Therefore, the aim of this study was to clarify this relationship. In vitro, we stimulated chicken macrophage cells (HD11) with lipopolysaccharide, then analyzed the correlation between SPOP and IL1β or IL8 expression using quantitative real-time polymerase chain reaction (qRT-PCR). In vivo, we infected 7-days-old chickens with *Salmonella* Typhimurium, then analyzed the association between SPOP expression and the immune response, including IL1β and IL8 expression, IgA production, and bacterial loads. We found that SPOP may participate in the regulation of the immune response in macrophage cells. SPOP expression was negatively correlated with IL-1β and IL-8 expression both in vivo and in vitro. SPOP expression was also negatively related to bacterial loads and immunoglobulin (Ig) A production. These results indicate that SPOP may have important functions in the response to *Salmonella* infection.

## 1. Introduction

*Salmonella* are Gram-negative enteric pathogens that colonize in chickens and threaten human health via the consumption of contaminated meat or eggs [1]. Although several methods have been applied to control infections in the poultry industry, including antibiotics, improvement of breeding environments, sterilization and vaccination, and salmonellosis cases continue to occur [2]. Furthermore, the use of antibiotics not only increases the resistance of *Salmonella* but also leads to the release of antibiotic residues, posing a risk to food safety [3]. With the development of molecular biology techniques, more research has focused on the regulation of the immune response to *Salmonella* infection, including efforts to identify new immune-related gene markers and to develop chicken disease resistance breeding programs [4,5].

Innate immunity is the first line of host defense against invading pathogens and functions to limit infection and activate acquired immunity [6,7]. Toll-like receptor (TLR) signaling pathways, especially the TLR4-MyD88-dependent pathway, and nucleotide-binding oligomerization domain (NOD)-like receptor (NLR) signaling pathways play an essential role in defense against *Salmonella* infection [8,9,10]. TLR4 is known to sense extracellular lipopolysaccharide (LPS) from Gram-negative bacteria, whereas NLRs are known to sense intracellular bacteria [11,12]. The recognition of pathogen-associated molecular patterns by TLRs or NODs initiates signal transduction pathways that induce the expression of cytokines [13,14]. Research has revealed an association between TLR4-MyD88 dependent pathway-related gene expression and *Salmonella* infection. TLR4, MyD88, and interleukin (IL) 8 increase after *Salmonella* infection [9,15]. Compared with the leukocytes of resistant chickens, leukocytes of susceptible chickens express lower levels of TLR4 as a result of DNA methylation in the predicted promoter region of TLR4 [8]. The polymorphic locus G247A of TLR4 is significantly associated with resistance to *Salmonella* in specific pathogenic free (SPF) white leghorn chickens [16], and Nod1 expression is upregulated in blood and spleen after *Salmonella* infection [17].

In addition to the gene expression changes observed after infection with *Salmonella*, many researchers have focused on the crucial molecules related to immune regulation and *Salmonella* infection. Gga-miR-1306-5p is induced by *Salmonella* and increases the expression of proinflammatory cytokines by targeting Toll interacting protein (Tollip) [18]. Li et al. [5] found that the FoxO signaling pathway was strongly activated in susceptible chickens, and FoxO3 was identified as a potential marker for host resistance to *Salmonella* infection.

Speckle-type POZ (poxvirus and zinc finger) protein (SPOP) is a member of the POZ family [19]. SPOP is a specific adaptor of Cul3-based ubiquitin ligase, which promotes substrate ubiquitination and subsequent degradation by proteasomes [20]. It also participates in the regulation of multiple cellular processes and is involved in skeletal development and cancer progression [21,22]. Previous research shows that SPOP promotes the degradation of MyD88 through such interactions and thus influences systemic inflammation [23]. However, its role in the immune response against *Salmonella* is still uncertain. Therefore, the objective of this study was to assess the relationship between SPOP expression and the immune response against *Salmonella*.

## 2. Materials and Methods

### 2.1. Ethics Statement and Animal Breeding

All sample collections and treatments were performed in accordance with the guidelines of the Animal Ethics Committee of the Institute of Animal Sciences, Chinese Academy of Agricultural Sciences (IAS-CAAS, Beijing, China) (approval number: IASCAAS-AE20140615).

Jingxing yellow chickens were obtained from the Changping Experimental Base of the Institute of Animal Sciences (Beijing, China). After hatching, the birds were checked for the presence of *Salmonella* by culturing fecal samples in buffered peptone water overnight with shaking and spreading the samples on brilliant green agar for incubation at 37 °C for 18–24 h. Positive chickens were excluded from the study. Negative chickens were raised in separate cages at the experimental center of China Agricultural University (Beijing, China) and given access to feed (SPF grade, supplied by Beijing Keao Xieli Limited Company, Beijing, China) and water.

### 2.2. Bacterial Strains

The *Salmonella* Typhimurium strain (ST, 21484) was purchased from China Industrial Microbial Culture Preservation Center (Beijing, China). Bacteria were restored and cultured in Luria-Bertani Broth (Amresco, Washington, DC, USA) at 37 °C with shaking.

### 2.3. Experimental Infection and Sample Collection

ST was cultured for 12 h and concentrated in a centrifuge. The final number of colony-forming units (CFUs) was determined by plating serial dilutions. At 7 days of age, a total of 40 chickens were orally inoculated with 1 mL of a bacterial suspension containing 2.5 × 10^10^ CFU ST. In the control group, 40 chickens were given the same volume of saline. Birds were sacrificed 3 days after infection, and blood samples, livers, and spleens were collected. Blood was allowed to clot and serum was stored. The livers were for the measurement of bacterial loads. The spleens were placed in liquid nitrogen for later RNA extraction. In the later experiments, 16 chickens were randomly selected from each group for the measures of relative expression.

### 2.4. Measurement of IgA Concentrations and Bacterial Loads

Sixteen chickens in the infected group were used for the measure of immunoglobulin A (IgA) and bacteria loads. For IgA measurements, serum was diluted and measured according to the manufacturer’s instructions (Cusabio, Wuhan, China). In short, standards or serum diluent (100-fold dilution) with Horseradish Peroxidase (HRP) conjugated antibody for IgA were added to plate precoated with IgA. The competitive inhibition reaction was launched between with precoated IgA and IgA in samples. Following incubation and wash, color developed after the addition of substrate solution. The intensity of the color was measured by microplate reader. According to the standards, the IgA concentrations in the serum were calculated. For the determination of bacterial loads, the liver samples of same weight (about 0.1 g) and similar location were taken and ground in a machine, then diluted with saline and plated onto Bismuth Sulphite Agar (Aoboxing, Beijing, China). After incubation for 24 h at 37 °C, the number of colonies were counted to determine bacterial loads.

### 2.5. Cell Culture and LPS Stimulation

The chicken macrophage cell line HD11 was cultured in RPMI 1640 media supplemented with 10% fetal bovine serum (FBS), 10 mM 4-(2-hydroxyethyl)-1-piperazine ethanesulfonic acid (HEPES), 2 mM glutamine,1 mM sodium pyruvate, 0.1 mM nonessential amino acids, 5% chicken serum, 100 units/mL penicillin, 100 μg/mL streptomycin, and 5 × 10^−5^ M β-mercaptoethanol (all reagents from Thermo Fisher Scientific, Waltham, MA, USA) at 37 °C. LPS was obtained from Sigma-Aldrich (St. Louis, MI, USA). For LPS stimulation, HD11 cells were challenged with 100 ng/mL LPS and harvested at the indicated times for RNA extraction. Each group included three biological replicates.

### 2.6. RNA Extraction and Quantitative Real-Time Polymerase Chain Reaction

Total RNA was extracted from spleens using TRIzol reagent (Thermo Fisher Scientific, Waltham, MA, USA) according to the manufacturer’s instructions. Reverse transcription was performed using a Reverse Transcriptase Kit (Tian’gen, Beijing, China). Quantitative real-time polymerase chain reaction (qRT-PCR) was performed using SYBR Fast qPCR Master Mix (KAPA, Wilmington, MA, USA). Each reaction was performed in triplicate. The qRT-PCR parameters were as follows: 95 °C for 3 min, 40 cycles of 95 °C for 3 s, and 60 °C for 34 s. The primers for qRT-PCR are listed in Table 1. Relative transcript abundance for genes was normalized to β-actin using 2^−ΔΔCT^ method.

### 2.7. Statistical Analysis

All data were statistically analyzed by one-way ANOVA using GraphPad Prism version 6.0 (GraphPad Software, San Diego, CA, USA). Differences with *p* values < 0.05 were considered statistically significant. Results are expressed as mean ± standard deviation (SD).

## 3. Results

### 3.1. Association of SPOP Expression with the Immune Response in HD11 Cells

**Figure 1:**
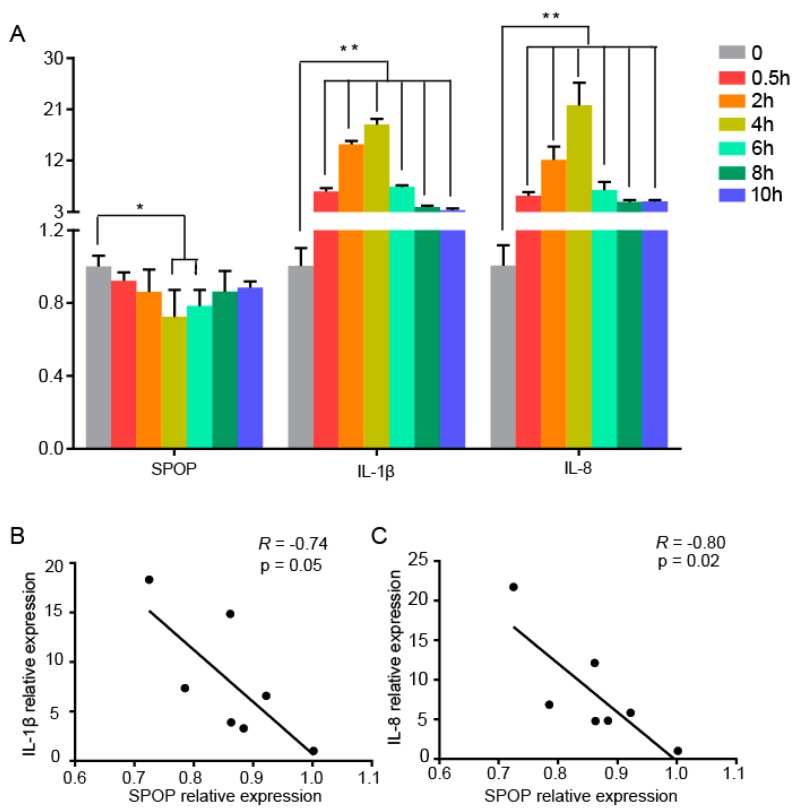
Speckle-type POZ (poxvirus and zinc finger) protein (SPOP) participated in the negative regulation of the immune response in cells. (**A**) HD11 cells were stimulated with lipopolysaccharide (LPS) for the indicated times. The cells were then harvested for RNA extraction. IL-1β, IL-8, and SPOP expression was measured using quantitative real-time polymerase chain reaction (qRT-PCR). Data were shown as means ± SD. * *p* < 0.05; ** *p* < 0.01 (**B**,**C**) Correlations between SPOP and IL-1β or IL-8 expression were analyzed using GraphPad Prism.

In order to determine the relationship between SPOP expression and the immune response in vitro, we stimulated the cells of the chicken macrophage HD11 line with bacterial substitute LPS for different lengths of time. We found that IL-1β and IL-8 increased significantly after challenge and peaked at 4 h (Figure 1A), indicating initiation of an immune response. Based on these results, we measured SPOP expression during LPS stimulation. SPOP expression decreased slightly and was significantly reduced at 6 h compared with controls, indicating that SPOP may be involved in the regulation of the immune response. To explore whether SPOP expression was related to IL-1β or IL-8 expression, the associations between their expression were investigated. As expected, SPOP expression was highly correlated with IL-1β (Figure 1B) and IL-8 (Figure 1C).

### 3.2. Changes in SPOP Expression after Salmonella Infection in Chickens

**Figure 2:**
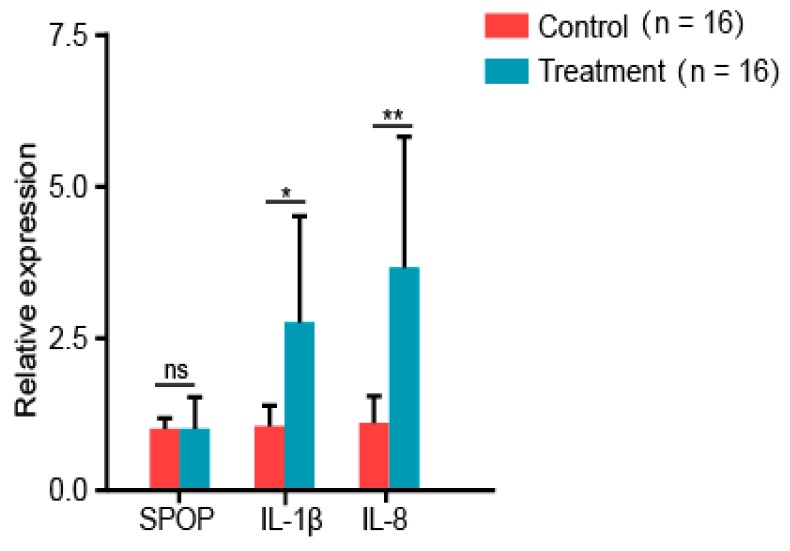
SPOP expression did not change after chickens were infected with *Salmonella*. RNA was extracted from Table 1. IL-8 and SPOP was measured using quantitative real-time polymerase chain reaction (qRT-PCR).

IL-1β and IL-8 were significantly higher in challenged birds compared to controls, indicating the occurrence of an immune response (Figure 2). Interestingly, SPOP mRNA levels remained unchanged (Figure 2). This was contradictory with results observed in cells, which may be related to the limited number of sample time points.

### 3.3. Association of SPOP Expression with the Immune Response After Salmonella Infection in Chickens

**Figure 3:**
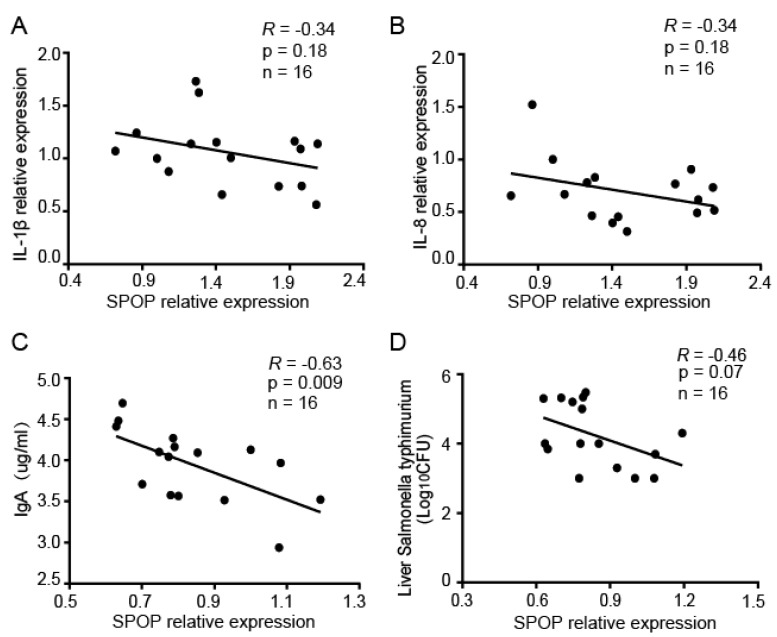
SPOP expression was negatively correlated with the immune response in chickens infected with *Salmonella*. (**A**,**B**) RNA was extracted from spleens and interleukin (IL) 1β, IL-8, and SPOP were measured using quantitative real-time polymerase chain reaction (qRT-PCR). Correlations between SPOP and (**A**) IL-1β or (**B**) IL-8 were analyzed. (**C**) Immunoglobulin (Ig) A concentrations in serum were measured using enzyme-linked immunosorbent assays (ELISAs), and correlations between SPOP expression and IgA concentrations were analyzed. (**D**) Liver samples of similar weights were ground, diluted, and plated onto Bismuth Sulphite Agar. After culturing for 24 h in an incubator, bacterial loads were counted.

After *Salmonella* infection, three immune-related traits were measured to assess the immune response, including IL-1β, IL-8 expression, IgA production, and bacterial loads. SPOP expression was negatively correlated with IL-1β, IL-8, and IgA production, bacterial loads (Figure 3A–D).

## 4. Discussion

The detection of *Salmonella* by pattern recognition receptors triggers an immune response, which includes the production of proinflammatory factors to limit bacterial replication. In chicken, TLR4-MyD88 dependent pathway plays an essential role in recognition of lipopolysaccharide (LPS) of Gram-negative bacteria and immune responses [24]. In this pathway, MyD88 links TLR4 with downstream factors. Upon combination of TLR4 with pathogens, MyD88 transduces signals to the IL-1R-associated kinase (IRAK) kinases, initiating the activation of downstream pathways, such as the transcription factor NF-κB and subsequent induction of a number of inflammatory response genes [25].

However, excessive inflammation and immune responses may lead to pathological damage, such as DNA damage or cancer [26,27], that is harmful to the host. To maintain tissue homeostasis, inflammatory responses must be terminated at the appropriate time, and this is achieved by short half-lives of proinflammatory mediators and anti-inflammatory signals [28]. Degradation of proinflammatory mediators including MyD88 by ubiquitination is a crucial mechanism to regulate the immune response. The E3 ubiquitin ligase Nrdp1 directly binds and polyubiquitinates MyD88, thereby enhancing host resistance to LPS-induced endotoxin shock [29]. CD11b promotes MyD88 degradation and negatively regulates TLR-triggered inflammatory responses via E3 ubiquitin ligase Cbl-b [30].

As an adaptor of Cul3-based ubiquitin ligase, SPOP is ubiquitously expressed, although in varied quantities, in different tissues [31]. It affects many biological processes by binding and triggering the ubiquitination and proteasomal degradation of target proteins [21,32]. Previous research indicates that SPOP binds with MyD88 and promotes the degradation [23]. MyD88 expression influences proinflammatory cytokines expression [28]. IL-1β and IL-8, two kinds of proinflammatory cytokines downstream of MyD88, were negatively correlated with SPOP expression in our research. Maybe SPOP functions upstream of these signaling pathways, specifically targeting MyD88 for ubiquitination and degradation and leading to less production of proinflammatory cytokines. While the inflammatory cytokines response is critical for the control of bacterial growth [33], excessive cytokines production can lead to endotoxic shock or sepsis-related deaths [34,35]. Previous study also shows that the bacteria loads and proinflammatory cytokines expression are lower in resistant chickens compared with those in susceptible chickens [5]. Our results showed when SPOP expression was lower, IL-1β and IL-8 expression and the bacteria loads were higher. This may be because overproduction of cytokines by immune cells and tissues renders chickens more susceptible to endotoxin shock and *Salmonella*–caused sepsis. IgA is one kind of major immunoglobulins that is involved in the development of the immune response to *Salmonella* [36]. SPOP was negatively correlated with IgA concentration, maybe because SPOP can normalize bacterial populations and immune response in *Salmonella* challenged chickens. So, SPOP may possess immune-protecting properties. However, whether SPOP regulates chicken resistance against *Salmonella* by specific regulation of MyD88, and whether SPOP influences chicken resistance against other pathogens, including Gram-positive bacteria and virus, needs to be investigated in future.

## 5. Conclusions

SPOP expression was negatively correlated with IL-1β and IL-8 mRNA expressions both in vivo and in vitro. Besides, when SPOP expression was greater, the bacteria load was lower. Taken together, our findings reveal that SPOP may participate in the regulation of the immune response and enhance resistance against *Salmonella*. Our findings on this novel function of SPOP provide a mechanistic basis for related future research.

## Figures and Tables

**Table 1:** Primers used for quantitative real-time polymerase chain reaction (qRT-PCR).

Name	Forward(5’-3′)	Reverse(5’-3′)
SPOP	AGGCTTGGATGAGGAGAGT	CGCTGGCTCTCCATTGCTT
IL-1β	GCATCAAGGGCTACAAGCTCT	CCAGGCGGTAGAAGATGAAG
IL-8	TCCTCCTGGTTTCAGCTGCT	GTGGATGAACTTAGAATGAGTG
β-actin	GAGAAATTGTGCGTGACATCA	CCTGAACCTCTCATTGCCA
